# Identification and Adulteration Evaluation of Rubiae Radix Et Rhizoma and Its Common Adulterants Based on LC-MS and Chemometrics

**DOI:** 10.3390/molecules30112385

**Published:** 2025-05-29

**Authors:** Lihui Zhang, Ting Han, Xianrui Wang, Yu Zhang, Jiating Zhang, Wenguang Jing, Minghua Li, Xianlong Cheng, Feng Wei

**Affiliations:** 1 Institute for Control of Traditional Chinese Medicine and Ethnic Medicine, National Institutes for Food and Drug Control, Beijing 102629, China; 13199634075@163.com (L.Z.); 13381100405@163.com (T.H.); niuyun006097@163.com (X.W.); zhangyu55505@163.com (Y.Z.); 18341441178@163.com (J.Z.); jingwenguang@nifdc.org.cn (W.J.);; 2 State Key Laboratory of Drug Regulatory Science, National Institutes for Food and Drug Control, Beijing 102629, China

**Keywords:** identification evaluation, chemometrics, Rubiae radix et rhizoma, *Rubia schumanniana* E. Pritz., *Rubia magna* P. G. Xiao, LC-MS

## Abstract

Background: *Rubia schumanniana* E. Pritz. (RSP) and *Rubia magna* P. G. Xiao (RMP) are common adulterants of Rubiae radix et rhizoma (RRR). To realize RRR adulteration analysis, this paper conducted an identification evaluation of RRR based on liquid chromatography–mass spectrometry (LC-MS). Methods: After LC-MS analysis and data conversion, the ion intersections were taken from different batches of RSP, RMP, and RRR. Then, the proprietary ions of three herbs were obtained by taking the de-intersection. Finally, the top-*n* ions were treated as the “ion identity” of RSP, RMP, and RRR for matching test samples to obtain the recognition index (RI) and combined chemometrics for analysis. Results: The identification evaluation of RRR can be realized based on the “ion identity”, even if 3% RSP (or RMP) in RRR samples can still be recognized. Combined with chemometric analysis, three market samples were identified as adulterants, proving that identification evaluation based on “ion identity” is reasonable. Conclusions: The “ion identity” based on LC-MS, which helps in realizing RRR identification evaluation. It has important reference significance for RRR analysis.

## 1. Introduction

Rubiae radix et rhizoma (RRR), known as “Qian Cao” in China, can cool blood, stop bleeding and remove blood stasis [1]. It can be traced back to Shennong’s Classic of the Materia Medica and has been used for thousands of years [2,3]. As far as varietal origin is concerned, the 2020 edition of Ch.P stipulates that RRR is the dried root and rhizome of *Rubia cordifolia* L, a Rubiaceae plant [3]. Strictly speaking, *Rubia cordifolia* L is the legal and only source of RRR, and the other varieties are fakes, of which *Rubia schumanniana* E. Pritz. (RSP) and *Rubia magna* P. G. Xiao (RMP) are the most common adulterants. However, to reduce costs and gain illegal benefits, unscrupulous traders often sell RSP and RMP as RRR or adulterate them into RRR, which seriously jeopardizes the interests of consumers and even endangers lives. Therefore, it is necessary to strengthen RRR identification evaluation.

To this end, Zhao et al. constructed the complete chloroplast genome of RRR [4]. Based on morphological and microscopic characteristics, researchers identified RRR, RMP and RSP, and the results showed that RMP has few fine roots and the wood is lighter in colour, while the wood part of the section of RRR is mainly yellow-red in colour [5,6,7]. Li et al. determined the contents of alizarin, lucidin, xanthopurpurin, purpurin, 6-hydroxyrubiadin, chrysophanol, and mollugin, and combined multivariate statistical analysis to construct a partial least squares discriminant analysis (PLS-DA) model, which can differentiate RRR, RMP, and RSP [8]. All the above studies help improve RRR quality control and market regulation. However, these studies focus more on species differentiation, and it is not ideal to identify whether the RRR is adulterated with RMP or RSP. Therefore, to address the above issues, RRR adulterant evaluation was realized based on LC-MS and “ion identity”.

Due to the characteristics of high sensitivity and high accuracy, LC-MS technology has been widely used in the identification and analysis of traditional Chinese medicine (TCM) [9,10,11]. Pu Y et al. used LC-MS technology to determine lucidin content and the results showed good linearity in the range of 0.005–2.64 ng and the average recovery rate was 97.17%; lucidin can be used for RRR identification and quality control [12]. Zheng et al. developed an effective ultra-high-performance liquid chromatography coupled with quadrupole time-of-flight tandem mass spectrometry (UHPLC/Q-TOF/MS) method to analyze the chemical constituents in rat plasma and urine after the oral administration of RRR extract and the results showed alizarin-1-O-β-glucuronide and purpurin-3-O-β-glucuronide were identified in rat plasma, which is helpful for RRR metabolic analysis and mechanisms of action [13]. Humbare et al. extracted RRR with methanol as the solvent, and identified more than 100 chemical constituents, such as 6-methoxygenic acid, Rubiprasin A, and 1-hydroxy-2-methyl anthraquinone, by UPLC-UV-MS, which is helpful for the identification and analysis of RRR [14]. Hu et al. explored the UHPLC/Q-TOF/MS method to characterize 45 quinones and quantify 24 quinones from Rubia plants and used chemometric methods to evaluate the chemical relationship between Rubia samples according to the composition quinones, which is helpful for Rubia species quality control and identification [15]. Zhang et al. established the UPLC-QQQ-MS/MS method, and qualitatively and quantitatively analyzed 14 kinds of cyclic peptide compounds in 20 Rubia plants; combined with chemometrics, a classification model of Rubia species was established, which provided a reference for the identification of RRR and its adulterants [16]. On the other hand, “ion identity” has been established based on the ordered array of high-precision mass spectrometry data, which have better specificity than single ion equivalents and contributes to the identification evaluation of RRR [17].

In this research, considering the high sensitivity and accuracy of LC-MS and the high-level specificity of “ion identity”, LC-MS was used to analyze RRR, RSP and RMP. After data conversion, the ion intersections were taken from different batches of RSP, RMP, and RRR. Then, the proprietary ions of three herbs were obtained by taking the de-intersection. Finally, the top *n* ions were treated as the “ion identity” of RSP, RMP, and RRR for matching test samples to obtain the recognition index (RI) and combined chemometrics for analysis.

## 2. Results

### 2.1. LC-MS Analysis

**Figure 1:**
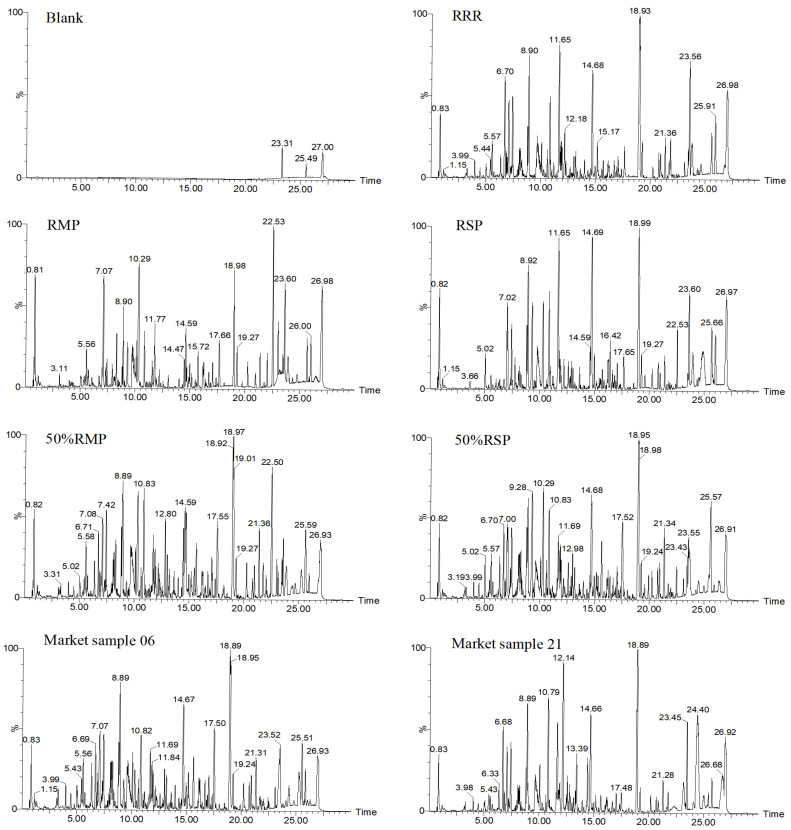
The base peak chromatograms of RRR, RMP, RSP, adulterants and market herbs.

Figure 1 shows the LC-MS results. Methanol (blank) displays virtually no assay interference. The RRR, RMP and RSP chromatograms had certain similarities, especially RRR and RSP. In other words, it is difficult to realize the identification of varieties when only relying on the comparison of base peak chromatograms, let alone adulterants. Therefore, we try to realize identification evaluation based on the LC-MS “ion identity” of RRR, RSP and RMP [17].

### 2.2. Data Processing and “Ion Identity”

The QI (2.4.69) was used for data conversion [18]. Table 1 shows the ion number of RRR, RMP, and RSP, which was used to obtain the “ion identity”.

RRR, RMP and RSP have different numbers of ions (different chemical compositions), but different batches of the same herb must have the same chemical compositions. So, the “ion identities” of RRR, RMP, and RSP were extracted based on different batches of RRR, RMP, and RSP, as shown in Table 1. After training and extraction, the top 50 ions were considered the “ion identity”. Tables S1–S3 show the “ion identity” of RRR, RMP, and RSP.

### 2.3. Identification of RRR, RSP and RMP

Table 2 shows the RI results. The RIs were all no less than 82.0% for RRR, RMP, and RSP samples, compared with their own “ion identities”; meanwhile, the RIs were all no higher than 6.00% for RRR, RMP, and RSP samples compared with their non-self “ion identities”, which is more than a 13-fold difference. Further, Table 3 shows the results of the nonparametric test (non-normal distribution); *p* = 0.004 < 0.01 showed that there were significant differences. The above results show that the “ion identities” of the three herbs have a certain specificity.

### 2.4. Adulteration Evaluation of RRR, RSP and RMP

#### 2.4.1. Evaluation of Adulterants

Table 4 shows the RI results of RRR and RMP adulterants. As the proportion of RMPs in adulterants increased from 3% to 100%, the RIs that compared with the RMP “ion identity” also showed an increasing trend, in which 3% RMP has the smallest RI (6.00%). At the same time, the RI of 0% RMP is only 0.00%, and the RI of 100% RMP is 96.0%. On the other hand, from 0% to 50% RMP, the RIs of adulterants are at least 90.0% compared to the RRR “ion identity”. However, the RI of 100% RMP is only 0.00% compared to the RRR “ion identity”. This shows that RMP adulteration identification based on RMP “ion identity” can be achieved without affecting RRR identification. It is worth noting that the MC of 20% RMP is 106% compared to the RRR “ion identity”. It is greater than 100% because the data bias (systematic error) leads to multiple sample ions being able to match single ions in the “ion identity” simultaneously. For example, the *m*/*z* 231.070 [M + H]^+^ and 231.057 [M + 2H]^2+^ in 20% RMP can match *m*/*z* 231.066 in RRR’s “ion identity”. In addition, the RIs of adulterants are no higher than 4.00% compared with the RSP “ion identity”.

Table 5 shows the RI results of RRR and RSP adulterants. Similarly, compared to the RSP “ion identity”, from 3% RMP to 100% RMP, the RIs of adulterants are no less than 12.0% when the RI of 0% RSP is only 0.00%. At the same time, this does not affect RRR identification based on the RRR “ion identity”. Except for 100% RSP (7.00%), the RIs of the remaining adulterants are at least 96.0%. In addition, the RIs of adulterants are no higher than 4.00% compared to the RMP “ion identity”.

The above results show that the “ion identities” of RRR, RSP, and RMP have certain specificity, which helps realize the identification of RRR, RSP and RMP. Further, considering the limit of 3% impurities, and in combination with Table 4 and Table 5, the detection threshold for RI based on the RMP “ion identity” was set to 6.00% (3% RMP). In the same way, the detection threshold for RI based on the RSP “ion identity” is 12.0% (3% RSP). In other words, based on the RI of 3% adulterated positive samples, if the RI is greater than the threshold set above, we can conclude that the sample is adulterated with RMP or RSP.

#### 2.4.2. Adulteration Evaluation of Market Samples

We carried out adulteration evaluation on 25 market RRR samples (MRS). Table 6 shows the RI results. The RIs of MRS20 and MRS24 were 58.0% and 40.0%, respectively, when compared to the RMP “ion identity”, which is much greater than the detection threshold of RMP (6.00%). Therefore, there is good reason to believe that samples MRS20 and MRS24 were adulterated with RMP based on the “ion identity” and RI. Compared to the RSP “ion identity”, the RIs of MRS05 and MRS24 were 78.0% and 58.0%, respectively, which is at least 4.8 times the detection threshold of RSP (12.0%). So, the samples MRS05 and MRS24 can be regarded as adulterated with RSP. The RIs of MRS were no less than 62.0% compared to the RRR “ion identity”. In summary, sample MRS05 was adulterated with RSP, while sample MRS20 was adulterated with RMP and sample MRS24 was adulterated with both RSP and RMP.

#### 2.4.3. Chemometric Analysis

**Figure 2:**
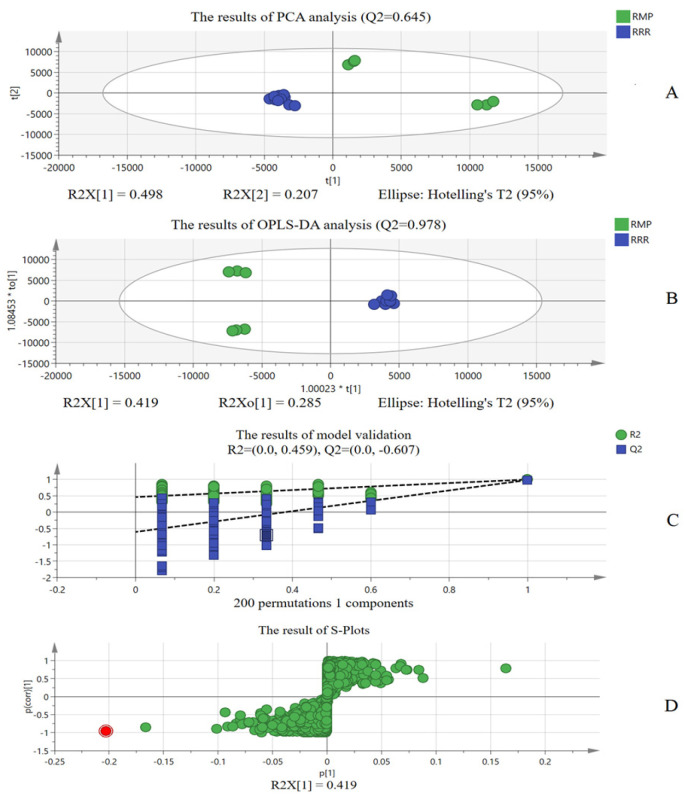
The results of chemometric analysis of RRR and RMP ((**A**) shows the results of PCA analysis; (**B**) shows the results of OPLS−DA analysis; (**C**) shows the results of model validation; (**D**) shows the results of S−Plots; Red dot: Different chemical compositions of RMP and RRR; Green dots: Chemical composition with no obvious difference).

MRS adulteration evaluation based only on “ion identity” was one-sided. Therefore, chemometric analysis was also used to explore differential ions for MRS adulteration evaluation. Figure 2 shows the results of the chemometric analysis of 11 RRRs and 6 RMPs. Figure 2A shows that RRR can be clearly distinguished from RMP using principal component analysis (PCA) with R2X = 0.705 and Q2 = 0.645, which indicates that RRR and RMP have different chemical components [19]. Figure 2B shows that RRR and RMP can also be effectively distinguished from each other using supervised orthogonal partial least squares discriminant analysis (OPLS-DA) with Q2 = 0.978 [20,21]. At the same time, the Q2 = (0.0, −0.607) in Figure 2C shows that the analysis is reasonably reliable. Figure 2D shows that the red data point in the lower left of the “S-curve” represents the differential component that is highly abundant in the RMP but at baseline levels in the RRR [22]. The red data point is the chemical marker A−(t*_R_* 14.46 min_*m*/*z* 459.1488).

**Figure 3:**
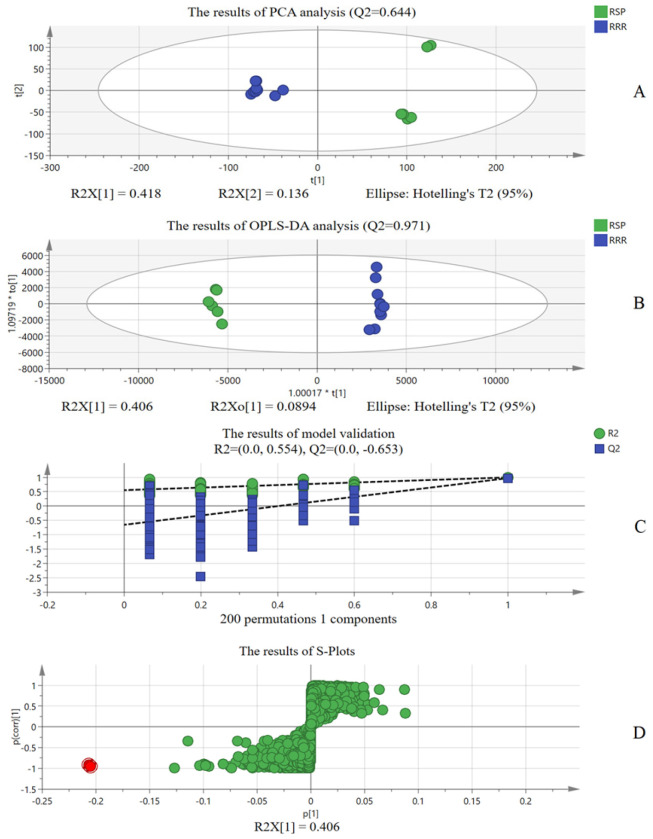
The results of chemometric analysis of RRR and RSP ((**A**) shows the results of PCA analysis; (**B**) shows the results of OPLS−DA analysis; (**C**) shows the results of model validation; (**D**) shows the results of S−Plots; Red dot: Different chemical compositions of RMP and RRR; Green dots: Chemical composition with no obvious difference).

Likewise, mass spectrometry of 11 RRRs and 6 RSPs was converted into data pairs to conduct chemometric analysis; the results are shown in Figure 3. We can obtain RSP’s chemical marker B−(*t_R_* 9.29 min_*m/z* 815.3594). Unfortunately, we could not identify exactly what compounds A and B are. But it does not matter; it can also be used to assist in verification.

Figures S1 and S2 show the extraction results of chemical markers A and B. The chemical component A can be extracted from RMP, adulterants of RRR and RMP, and MRS20 and MRS24 samples; their ionic strengths are all higher than 1.0 × 10^4^, but at baseline levels in the RRR samples, 0% RRR sample and remaining MRS samples. The chemical component B existed in the RSP samples, adulterants of RRR and RSP, and MRS05 and MRS24 samples; their ionic strengths are all higher than 1.2 × 10^6^, but at baseline levels in the RRR samples, 0% RRR sample and remaining MRS samples. The above results indicate that the MRS05 and MRS20 are indeed adulterated with RSP and RMP, respectively, and the MRS24 is adulterated with both RSP and RMP. On the other hand, the chemometric analysis proved that adulteration evaluation based on the “ion identity” of RRR, RMP and RSP is reliable.

## 3. Discussion

In this paper, the “ion identity” based on LC-MS was proposed to conduct RRR identification evaluation. Firstly, LC-MS was optimized to obtain MS data [23,24,25,26,27]. We examined the ESI^+^ and ESI^−^ mode. Samples have more data in the positive mode. The collision voltage was optimized for 10~40 V. In terms of extraction solvents, methanol had the best extraction results. Then, the deviations for *t_R_* and *m*/*z* were optimized in the process of acquiring the “ion identity”. The *t_R_* drifted but did not exceed 0.1 min. On the other hand, we comprehensively compare the “ion identity” cases when the Δ*m*/*z* ≤ 0, 0.01, and 0.05 Da. The results showed that when Δ*m*/*z* ≤ 0.01 Da, this ensures sufficient precision, as well as allowing us to screen further based on ionic strength. However, the shared ions cannot be acquired when Δ*m*/*z* ≤ 0.00 Da and if Δ*m*/*z* ≤ 0.05 Da, there are more ion data, but larger deviation will easily lead to false positive results. Finally, the ion number in the “ion identity” is equally important for RRR identification evaluation. Therefore, we examined the matching situation when 50, 100, and 150 ions were output as their respective “ion identities”. As shown in Table 7, as the ion number in the “ion identity” increases, the RIs of the matches between RRR11, RMP06, and RSP06 and their own “ion identity” decreases, except for the RRR10 sample. Therefore, to take care of the matching between RMP, RSP and the “ion identity”, we finally chose to output the top 50 ions as their respective “ion identities”, which do not affect the matching of RRR simultaneously. If all ions are regarded as “ion identities” for matching identification, the matching effect becomes poor. Not all ions are valid data, some ions are invalid and jumbled, and the ion number in different herbs is different, which leads to the loss of comparative significance of RI. Reducing the number of ions in the “ion identity” to remove invalid data and focusing on high-abundance and highly specific ions can improve the matching accuracy.

As for RRR market samples, identification evaluation based only on the “ion identity” of LC-MS is not sufficient. Therefore, combined with chemometric analysis, the differential ions were extracted for adulteration identification. “Ion identity” based on LC-MS and chemometrics based on LC-MS combine and confirm each other, which fully proves adulteration in the RRR market samples and makes the results more reliable. On the other hand, chemometrics also proved that identification evaluation based on the “ion identity” of LC-MS was reliable. Moreover, compared with previous studies [17,18], the present study focused on the adulteration of same-family herbs, which are more difficult to identify compared to herbs with great differences. The research also fully considered the differential chemical compositions of different herbs and used this to construct an ordered quantized matrix of chemical compositions according to the “ion identity”, which has more specificity than a single or a few chemical components. RRR identification evaluation based on “ion identity” uses more accurate high-resolution mass spectrometry data to realize information and automatic identification and analysis. Compared with traditional microscopic analysis and data modeling analysis, the analysis method based on “ion identity” is more accurate, fast and reliable.

As for the detection thresholds of RMP and RSP adulteration, these are different from ROC and confusion matrix evaluation based on data modeling and identification. The detection thresholds of RMP and RSP were based on the RI test result of 3% adulterated positive samples. Therefore, in each subsequent matching identification, it is necessary to prepare such a 3% positive adulterated sample as a reference to determine the RI detection threshold. For the samples whose matching results are very close to the detection threshold, they can be further extracted and verified by the differential ions analyzed by chemometrics.

In this study, RRR identification evaluation was conducted based on the “ion identity” of LC-MS and chemometrics. However, there are still some regrets: (1) Although the samples involve different producing areas and are collected in person, the sample size is indeed limited, which can only meet the requirements of a preliminary exploration. (2) Although it does not affect the final evaluation, and fully proves the rationality and reliability of analysis based on “ion identity”, chemometric analysis, based on a comparison between existing chemical substance standards and references, fails to clarify what compounds the different chemical components A and B are. Therefore, the sample size could be increased to carry out analysis and explore these proprietary chemical compositions.

## 4. Materials and Methods

### 4.1. Herbs, Laboratory Consumables and Instrument

Totals of 11 RRRs, 6 RSPs and 6 RMPs were collected from different places. The above herbs were identified by Xianlong Cheng to meet the variety requirements. The 14 homemade positive mixed samples included 0% RMP, 3% RMP, 5% RMP, 10% RMP, 20% RMP, 50% RMP, and 100% RMP, and 0% RSP, 3% RSP, 5% RSP, 10% RSP, 20% RSP, 50% RSP, and 100% RSP [18,27]. In addition, AG herbal market provided 24 batches of market RRR materials. Table S4 shows the details on 61 batches of herbal materials. An ME-303 Electronic Balance was purchased from Plisys International Trading (Shanghai, China) Co., Ltd. We also used Waters H-Class UPLC and Waters G2-XS Q-TOF coupling instruments (Waters technology Co., Ltd., Milford, MA, USA). Milli water instruments were used to prepare deionized water. Thermo Honeywell Trading Co., Ltd. (Charlotte, NC, USA) provided methanol (Lot: E1058-US), formic acid (Lot: L1670) and Acetonitrile (Lot: Y5BA1H).

### 4.2. LC-MS Experimental Parameters

The Waters Acquity UHPLC-BEH C_18_ column (2.1 mm × 100 mm, 1.7 μm, Lot: 186002352, Waters technology Co., Ltd., Milford, MA, USA) was used for chromatographic separations. Column temperature: 35 °C; mobile phase velocity: 0.3 mL/min; sample load: 2 μL. Table 8 shows the gradient change in the mobile phase. ESI^+^, ESI^−^ and MS^E^ modes were adopted in MS analysis. *m*/*z*: 100 to 1200 Da; scan time: 0.1 s; source and desolvation temperature: 120 °C and 400 °C; desolvation and cone flow: 700 and 60 L/h; capillary and cracking: 3000 V (+), 2500 V (−) and 10~40 V. The MS resolution was set to 3.0 × 10^4^.

### 4.3. Sample Pretreatment

Totals of 11 RRRs, 6 RMPs and 6 RSPs were pulverized and passed through a No.3 sieve. According to different herbs, the reference samples of RRR, RSP and RSM were, respectively, prepared by mixing the powders of the respective batches in equal amounts. Reference samples of RRR, RSM and RSP were then used to prepare mixed positive samples with different ratios of adulteration (0%, 3%, 5%, 10%, 20%, 50% and 100%) [27].

We accurately weighed 1.0 g of various analyzed sample powders, added 50.00 mL methanol and sonicated for 0.50 h (400 W; 40 kHz). Finally, these were filtered using a 0.22 μm filter membrane to obtain test samples [8,14,18].

### 4.4. Data Pretreatment and Analysis Flow

**Figure 4:**
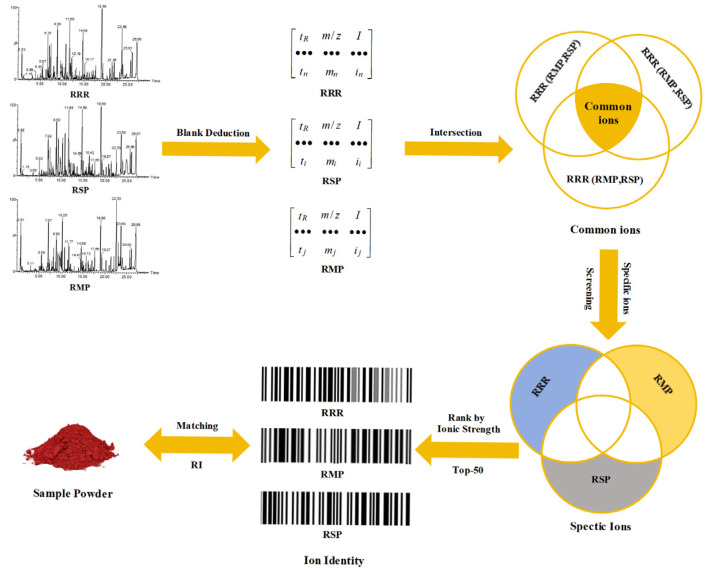
The identification flow based on the “ion identity”.

QI workstation was used to convert the MS information into [*t_R_*-*m*/*z*-I] data [28]. As shown in Figure 4, after data conversion, the intersections of ions were taken from different batches of RSP, RMP, and RRR. Then, the specific ions of three herbs were obtained by taking the de-intersection. The top 50 ions were treated as the “ion identity” of RSP, RMP, and RRR for matching test samples to obtain the recognition index (RI). In the above steps, if the ion data have similar *t_R_* (Δ*t_R_* ≤ 0.10 min) and *m*/*z* (Δ*m*/*z* ≤ 0.01 Da), they are regarded as duplicate or eliminated as interference ion data.

## 5. Conclusions

In this paper, the “ion identity”, based on LC-MS and chemometrics, was successfully applied for RRR analysis. Even if 3% RSP (or RMP) in RRR samples can still be recognized and combined with chemometric analysis, three market samples were identified as adulterants, proving that identification evaluation based on the “ion identity” is reasonable. The “ion identity” based on LC-MS, which helps to realize RRR identification evaluation and has important reference significance for RRR analysis.

## Figures and Tables

**Table 1:** The ion number in herbs used to extract “ion identity”.

Herbs	Batch	Number	Herbs	Batch	Number	Herbs	Batch	Number
RRR	RRR01	20,229	RMP	RMP01	21,063	RSP	RSP01	21,106
	RRR02	19,575		RMP02	21,721		RSP02	21,373
	RRR03	21,288		RMP03	21,137		RSP03	20,319
	RRR04	20,996		RMP04	20,425		RSP04	19,876
	RRR05	20,721		RMP05	19,857		RSP05	20,591
	RRR06	20,340						
	RRR07	20,603						
	RRR08	19,748						
	RRR09	20,344						

**Table 2:** The RI results of RRR, RMP, and RSP test samples.

Herbs	Batches	Match Ions	Ion Identity of RRR, RMP or RSP	RI (%)
RRR	RRR10	47	50-RRR	94.0
		0	50-RMP	0.00
		0	50-RSP	0.00
	RRR11	41	50-RRR	82.0
		0	50-RMP	0.00
		1	50-RSP	2.00
RMP	RMP06	46	50-RMP	92.0
		0	50-RRR	0.00
		0	50-RSP	0.00
RSP	RSP06	48	50-RSP	96.0
		0	50-RRR	0.00
		3	50-RMP	6.00

**Table 3:** The results of the nonparametric test.

Index	Median (P_25_, P_75_) 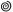		Mann Whitney–U	Mann Whitney–Z	*p*
	Group I (*n* = 4)	Group II (*n* = 8)			
RI (%)	93.0 (84.5, 95.5)	0.00 (0.0, 1.5)	0.000	−2.901	0.004 **

* *p* < 0.05, ** *p* < 0.01; Group I: the RIs compared with their own “ion identity”; Group II: the RIs compared with their non-self “ion identity”.

**Table 4:** The RI results of RRR and RMP adulterants.

Adulterants	“Ion Identity” of RMP	“Ion Identity” of RRR	“Ion Identity” of RSP
0% RMP	0.00%	96.0%	0.00%
3% RMP	6.00%	98.0%	4.00%
5% RMP	16.0%	100%	2.00%
10% RMP	24.0%	100%	2.00%
20% RMP	36.0%	106%	2.00%
50% RMP	70.0%	96.0%	0.00%
100% RMP	96.0%	0.00%	0.00%

**Table 5:** The RI results of RRR and RSP adulterants.

Adulterants	“Ion Identity” of RSP	“Ion Identity” of RRR	“Ion Identity” of RMP
0% RSP	0.00%	94.0%	0.00%
3% RSP	12.0%	96.0%	2.00%
5% RSP	16.0%	98.0%	4.00%
10% RSP	20.0%	102%	2.00%
20% RSP	54.0%	102%	4.00%
50% RSP	78.0%	96.0%	2.00%
100% RSP	86.0%	7.00%	2.00%

**Table 6:** The RI results of market RRR samples.

MRS	“Ion Identity” of RMP	“Ion Identity” of RRR	“Ion Identity” of RSP
MRS01	0.00%	76.0%	4.00%
MRS02	0.00%	78.0%	4.00%
MRS03	0.00%	82.0%	2.00%
MRS04	0.00%	82.0%	6.00%
MRS05	2.00%	96.0%	78.0%
MRS06	0.00%	80.0%	6.00%
MRS07	2.00%	72.0%	2.00%
MRS08	0.00%	74.0%	4.00%
MRS09	4.00%	70.0%	8.00%
MRS10	2.00%	72.0%	8.00%
MRS11	0.00%	78.0%	4.00%
MRS12	2.00%	76.0%	6.00%
MRS13	2.00%	82.0%	4.00%
MRS14	2.00%	72.0%	2.00%
MRS15	2.00%	74.0%	6.00%
MRS16	2.00%	74.0%	4.00%
MRS17	2.00%	62.0%	4.00%
MRS18	2.00%	74.0%	4.00%
MRS19	0.00%	82.0%	2.00%
MRS20	58.0%	88.0%	0.00%
MRS21	0.00%	78.0%	4.00%
MRS22	4.00%	68.0%	6.00%
MRS23	2.00%	72.0%	2.00%
MRS24	40.0%	62.0%	58.0%
MRS25	0.00%	78.0%	4.00%

**Table 7:** The RI results when the “ion identity” contains 50, 100, and 150 ions.

Herbs	Batch	50 Ions	100 Ions	150 Ions
RRR	RRR10	94.0%	95.0%	93.0%
	RRR11	82.0%	78.0%	74.0%
RMP	RMP06	92.0%	81.0%	68.0%
RSP	RSP06	96.0%	94.0%	91.0%

**Table 8:** The gradient change in the mobile phase.

Time (min)	Acetonitrile (%)	0.1% FA Water (%)	Flow (L/min)
0.00	5	95	0.3
0.30	5	95	0.3
23.00	95	5	0.3
26.00	95	5	0.3
26.10	5	95	0.3
30.00	5	95	0.3

## Data Availability

Data information can be obtained in this paper or by contacting the corresponding author.

## Supplementary Materials

The following supporting information can be downloaded at: https://www.mdpi.com/article/10.3390/molecules30112385/s1, Table S1: The “ion identity” of Rubiae radix et rhizoma (RRR); Table S2: The “ion identity” of Rubia schumanniana E. Pritz. (RSP); Table S3: The “ion identity” of Rubia magna P. G. Xiao (RMP); Table S4: The detailed information of herbal materials; Figure S1: The detection situation of marked ions of RMP (chemical composition A); Figure S2: The detection situation of marked ions of RSP (chemical composition B).
