# Supplementary material for: Identification and Adulteration Evaluation of Rubiae Radix Et Rhizoma and Its Common Adulterants Based on LC-MS and Chemometrics
Source: Molecules. 2025 May 29;30(11):2385. doi: 10.3390/molecules30112385 (PMC12156058; doi:10.3390/molecules30112385)
Supplement: Supplementary file 1 [file molecules-30-02385-s001.zip › molecules-3605911-supplementary.pdf]

Identification Evaluation of Rubiae Radix et Rhizoma based on LC-MS

Lihui Zhang[1,2†], Ting Han[1,2†], Xianrui Wang[1,2†], Yu Zhang[1,2], Jiating Zhang[1,2], Wenguang Jing[1,2], Minghua Li[1,2], Xianlong Cheng[1,2,*], Feng Wei[1,2,*]

Table S1 The "ion identity" of Rubiae radix et rhizoma (RRR)

t_R	*m/z*	I	t_R	*m/z*	I
5.49	581.226	157722	10.81	417.154	16855
10.44	662.392	131666	13.29	315.087	16716
10.03	628.406	71969	6.20	561.171	16715
9.76	588.318	64066	6.01	765.256	16578
25.74	975.544	56948	7.25	576.390	15098
12.32	419.170	44217	8.60	717.417	14626
8.82	592.310	39923	12.33	1073.375	13571
11.73	287.130	38942	11.50	285.113	13406
8.26	558.327	32935	22.07	339.290	13309
19.59	593.145	31527	12.32	856.306	12897
22.50	660.473	29773	7.25	1032.537	12397
15.95	395.222	29362	19.45	441.338	12176
7.58	501.344	27643	4.70	717.335	12066
18.92	1038.655	26853	9.90	614.391	11913
10.33	648.376	26592	6.70	253.190	11892
10.96	387.144	24411	7.72	544.313	11466
12.32	341.139	24184	10.21	770.235	11423
12.32	864.292	23116	12.32	473.136	11294
9.60	457.127	21298	5.68	478.171	11010
5.50	598.253	20903	20.05	876.258	10954
18.47	582.172	20794	6.87	1086.308	10835
9.59	441.153	19339	10.46	549.193	10738
12.30	514.116	19206	15.07	533.121	10717
9.18	231.066	18436	9.28	844.274	10685
8.99	457.126	18121	4.45	1112.258	10550

Table S2 The "ion identity" of Rubia schumanniana E. Pritz. (RSP)

t_R	*m/z*	I	t_R	*m/z*	I
24.85	708.511	560629	11.43	1017.383	8642
10.07	262.254	129950	14.72	1155.396	8330
8.49	276.235	111269	21.36	905.310	8226
25.49	916.586	61531	11.98	583.190	7905
13.13	471.348	53408	18.45	903.317	7762
6.80	306.244	42209	12.96	623.226	7664
17.71	1055.399	36429	19.23	921.305	7500
23.27	497.359	34714	7.02	1048.192	7387
13.21	277.217	31861	7.00	903.277	7346
7.02	278.082	27764	14.20	997.358	7096
23.43	501.385	23405	14.98	729.328	6948
19.23	905.334	15105	19.61	890.264	6877
14.69	252.350	14530	21.89	962.269	6618
12.32	1031.401	13804	7.00	887.223	6419
16.22	699.145	13552	14.34	437.344	6378
19.04	1113.446	13516	15.10	1115.455	6079
16.80	1099.463	12499	16.42	687.359	5787
15.69	1115.458	11973	21.53	893.228	5640
15.16	611.117	11679	22.18	915.504	5571
9.30	815.360	11452	12.96	869.405	5503
16.11	993.271	11088	21.89	967.223	5482
12.72	523.174	10360	13.20	679.385	5245
25.77	875.230	9301	11.42	971.377	5175
24.57	635.383	8992	12.12	867.387	5131
15.52	1115.454	8841	17.03	754.206	5098

Table S3 The "ion identity" of Rubia magna P. G. Xiao (RMP)

t_R	*m/z*	I	t_R	*m/z*	I
2.87	311.233	109546	3.28	271.239	10539
14.46	715.170	70800	16.45	585.156	10337
21.77	720.450	53344	7.60	259.097	10235
13.88	627.110	37513	16.51	497.068	10183
4.15	280.156	34269	17.11	540.110	9765
11.04	327.161	30791	15.94	479.099	9340
13.55	285.076	30013	12.15	373.071	9336
4.89	297.256	29994	16.46	583.153	8508
14.59	531.094	28346	9.34	466.186	8338
14.46	292.088	27376	15.53	835.128	7327
10.13	653.974	27243	7.13	199.076	7309
25.16	663.387	22399	16.50	545.088	6986
23.20	579.370	21668	5.69	588.186	6210
12.05	471.087	16582	7.12	321.112	5869
17.47	657.139	15017	14.64	467.092	5770
14.46	459.149	14916	14.46	529.103	5520
11.75	517.093	13853	15.51	691.124	5328
14.46	276.094	13841	3.88	308.223	5088
12.51	785.196	13344	13.56	597.134	5046
19.39	582.167	13328	14.24	489.097	4962
9.30	643.158	12680	24.34	755.388	4933
17.08	900.478	12574	11.76	515.373	4896
24.77	483.087	11784	25.29	757.408	4878
16.36	579.114	11679	25.79	661.379	4667
14.46	707.184	10867	22.85	527.078	4496

Table S4 The detailed information of herbal materials

Herbal materials	Batch	Sample Source	Use	Place of origin
Rubiae radix et rhizoma	RRR01	National Institutes for Food and Drug Control	"ion identity"; mixed sample	Shanxi, China
Rubiae radix et rhizoma	RRR02	National Institutes for Food and Drug Control	Identification evaluation; mixed sample	Hebei, China
Rubiae radix et rhizoma	RRR03	National Institutes for Food and Drug Control	"ion identity"; mixed sample	Henan, China
Rubiae radix et rhizoma	RRR04	National Institutes for Food and Drug Control	"ion identity"; mixed sample	Hebei, China
Rubiae radix et rhizoma	RRR05	National Institutes for Food and Drug Control	"ion identity"; mixed sample	Shandong, China
Rubiae radix et rhizoma	RRR06	National Institutes for Food and Drug Control	"ion identity"; mixed sample	Jiangsu, China
Rubiae radix et rhizoma	RRR07	National Institutes for Food and Drug Control	"ion identity"; mixed sample	Gansu, China
Rubiae radix et rhizoma	RRR08	National Institutes for Food and Drug Control	"ion identity"; mixed sample	Gansu, China
Rubiae radix et rhizoma	RRR09	National Institutes for Food and Drug Control	Identification evaluation; mixed sample	Sichuan, China
Rubiae radix et rhizoma	RRR10	National Institutes for Food and Drug Control	"ion identity"; mixed sample	Hubei, China
Rubiae radix et rhizoma	RRR11	National Institutes for Food and Drug Control	"ion identity"; mixed sample	Xizang, China
Rubia schumanniana E. Pritz.	RSP01	Gansu Institutes for Food and Drug Control	"ion identity"; mixed sample	Hubei, China
Rubia schumanniana E. Pritz.	RSP02	Gansu Institutes for Food and Drug Control	Identification evaluation; mixed sample	Sichuan, China
Rubia schumanniana E. Pritz.	RSP03	Gansu Institutes for Food and Drug Control	"ion identity"; mixed sample	Yunnan, China
Rubia schumanniana E. Pritz.	RSP04	Gansu Institutes for Food and Drug Control	"ion identity"; mixed sample	Shanxi, China
Rubia schumanniana E. Pritz.	RSP05	Gansu Institutes for Food and Drug Control	"ion identity"; mixed sample	Sichuan, China
Rubia schumanniana E. Pritz.	RSP06	Gansu Institutes for Food and Drug Control	Identification evaluation; mixed sample	Guangxi, China
Rubia magna P. G. Xiao	RMP01	Gansu Institutes for Food and Drug Control	"ion identity"; mixed sample	Sichuan, China

Rubia magna P. G. Xiao	RMP02	Gansu Institutes for Food and Drug Control	"ion identity"; mixed sample	Guanxian, China
Rubia magna P. G. Xiao	RMP03	Gansu Institutes for Food and Drug Control	"ion identity"; mixed sample	Baoxing,China
Rubia magna P. G. Xiao	RMP04	Gansu Institutes for Food and Drug Control	"ion identity"; mixed sample	Tianquan, China
Rubia magna P. G. Xiao	RMP05	Gansu Institutes for Food and Drug Control	Identification evaluation; mixed sample	Sichuan, China
Rubia magna P. G. Xiao	RMP06	Gansu Institutes for Food and Drug Control	"ion identity"; mixed sample	Rongjing, China
0 % RMP	MIX01	self-preparation	Identification evaluation	—
3 % RMP	MIX02	self-preparation	Identification evaluation	—
5 % RMP	MIX03	self-preparation	Identification evaluation	—
10 % RMP	MIX04	self-preparation	Identification evaluation	—
20 % RMP	MIX05	self-preparation	Identification evaluation	—
50 % RMP	MIX06	self-preparation	Identification evaluation	—
100 % RMP	MIX07	self-preparation	Identification evaluation	—
0 % RSP	MIX08	self-preparation	Identification evaluation	—
3 % RSP	MIX09	self-preparation	Identification evaluation	—
5 % RSP	MIX10	self-preparation	Identification evaluation	—
10 % RSP	MIX11	self-preparation	Identification evaluation	—
20 % RSP	MIX12	self-preparation	Identification evaluation	—
50 % RSP	MIX13	self-preparation	Identification evaluation	—
100 % RSP	MIX14	self-preparation	Identification evaluation	—
Market RRR sample	MRS01	Herbal Markets	Adulterant evaluation	Gansu, China

Market RRR sample	MRS02	Herbal Markets	Adulterant evaluation	Gansu, China
Market RRR sample	MRS03	Herbal Markets	Adulterant evaluation	Gansu, China
Market RRR sample	MRS04	Herbal Markets	Adulterant evaluation	Anhui, China
Market RRR sample	MRS05	Herbal Markets	Adulterant evaluation	Anhui, China
Market RRR sample	MRS06	Herbal Markets	Adulterant evaluation	Anhui, China
Market RRR sample	MRS07	Herbal Markets	Adulterant evaluation	Zhejiang, China
Market RRR sample	MRS08	Herbal Markets	Adulterant evaluation	Zhejiang, China
Market RRR sample	MRS09	Herbal Markets	Adulterant evaluation	Zhejiang, China
Market RRR sample	MRS10	Herbal Markets	Adulterant evaluation	Xizang, China
Market RRR sample	MRS11	Herbal Markets	Adulterant evaluation	Xizang, China
Market RRR sample	MRS12	Herbal Markets	Adulterant evaluation	Xizang, China
Market RRR sample	MRS13	Herbal Markets	Adulterant evaluation	Xizang, China
Market RRR sample	MRS14	Herbal Markets	Adulterant evaluation	Xizang, China
Market RRR sample	MRS15	Herbal Markets	Adulterant evaluation	Shandong, China
Market RRR sample	MRS16	Herbal Markets	Adulterant evaluation	Shandong, China
Market RRR sample	MRS17	Herbal Markets	Adulterant evaluation	Hebei, China
Market RRR sample	MRS18	Herbal Markets	Adulterant evaluation	Hebei, China
Market RRR sample	MRS19	Herbal Markets	Adulterant evaluation	Hebei, China
Market RRR sample	MRS20	Herbal Markets	Adulterant evaluation	Hebei, China
Market RRR sample	MRS21	Herbal Markets	Adulterant evaluation	Hebei, China

RRR blind sample	MRS22	Herbal Markets	Adulterant evaluation	Hebei, China
RRR blind sample	MRS23	Herbal Markets	Adulterant evaluation	Sichuan, China
RRR blind sample	MRS24	Herbal Markets	Adulterant evaluation	Sichuan, China
RRR blind sample	MRS25	Herbal Markets	Adulterant evaluation	Sichuan, China

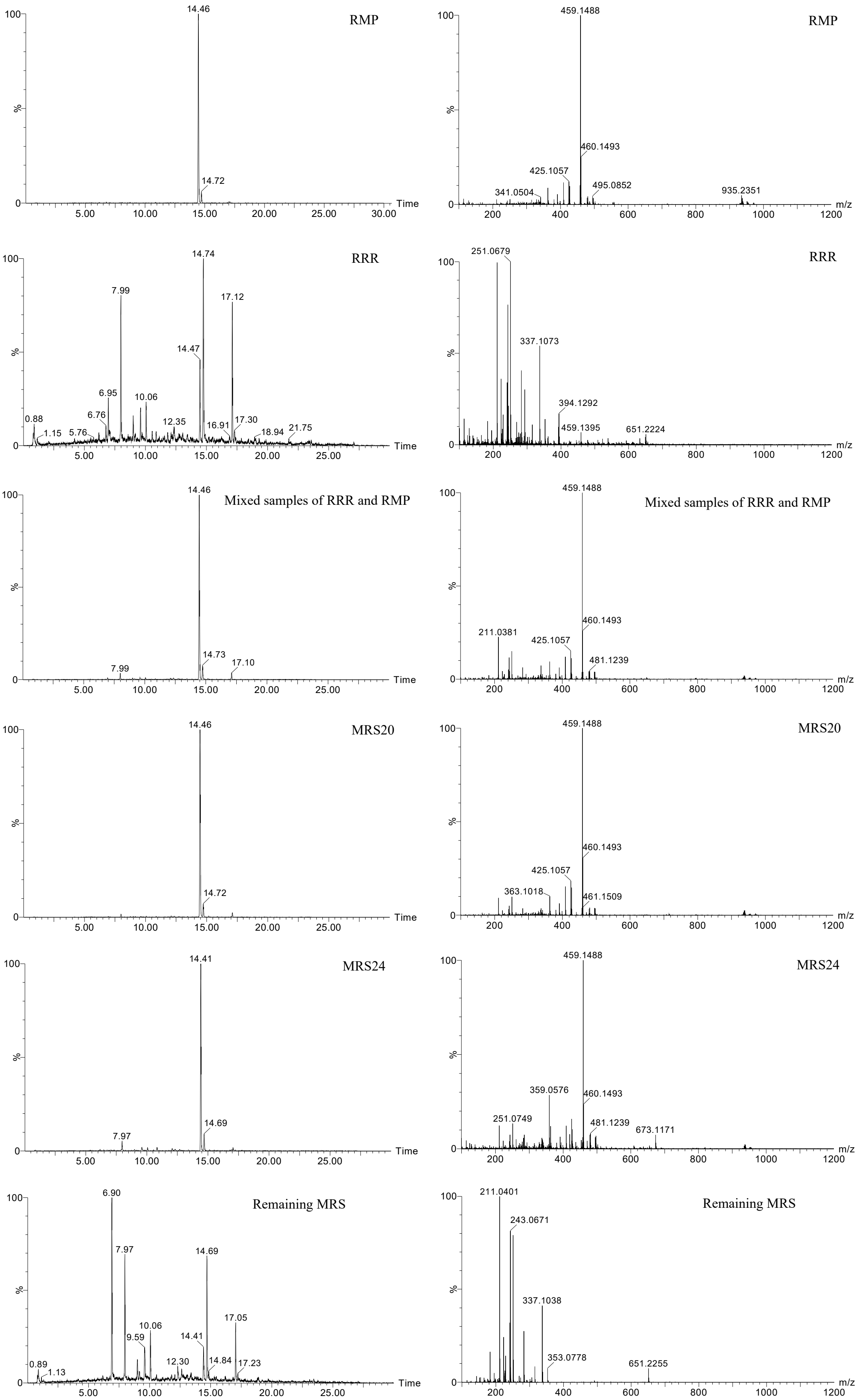

Figure S1. The detection situation of marked ions of RMP (chemical composition A)

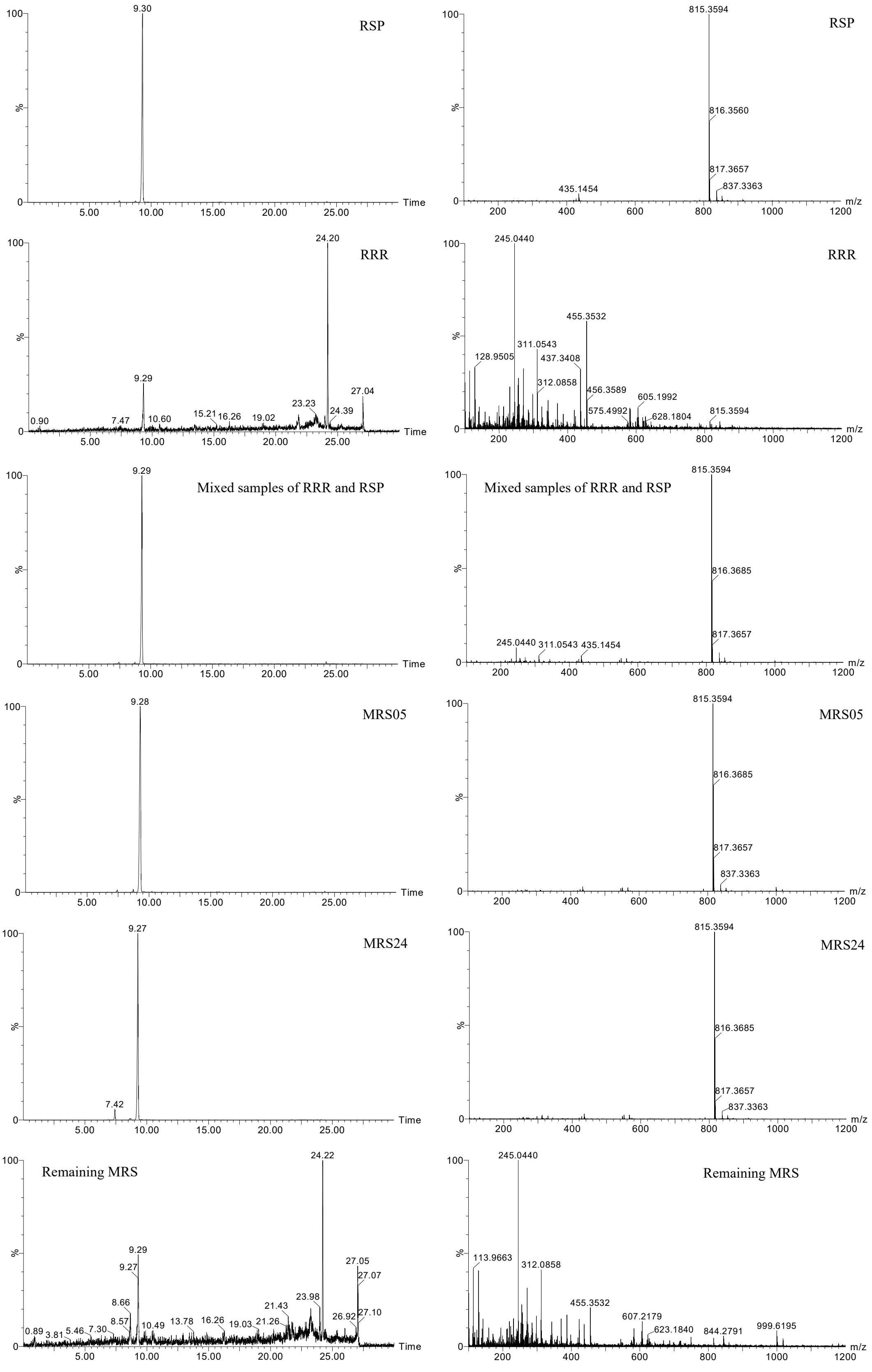

Figure S2. The detection situation of marked ions of RSP (chemical composition B)
